# RETRACTION Basel Khalil, Ibrahim Borghol, and Akhilanand Chaurasia, “Atypical Presentation of Polymicrobial Cervicofacial Necrotizing Fasciitis: An Extraordinary Odontogenic Infection in a Healthy Female Patient,” *Clinical Case Reports* 12, no. 9 (2024): e9414, https://doi.org/10.1002/ccr3.9382

**DOI:** 10.1002/ccr3.9704

**Published:** 2024-12-12

## Abstract

The article mentioned above, published online on August 27, 2024, on Wiley Online Library (wileyonlinelibrary.com), has been retracted by mutual agreement among the authors, the journal's Editor‐in‐Chief Dr. Charles Young, and John Wiley & Sons Ltd., UK. This decision was made following the second author's acknowledgment of an oversight in obtaining written consent from the patient for publication. The relevant data has since been redacted from the original manuscript. In addition, several scientific and logical inconsistencies were found in the article by the research integrity team.

The article mentioned above, published online on August 27, 2024, on Wiley Online Library (wileyonlinelibrary.com), has been retracted by mutual agreement among the authors, the journal's Editor‐in‐Chief Dr. Charles Young, and John Wiley & Sons Ltd., UK. This decision was made following the second author's acknowledgment of an oversight in obtaining written consent from the patient for publication. The relevant data has since been redacted from the original manuscript. In addition, several scientific and logical inconsistencies were found in the article by the research integrity team.

